# Increasing the yield of drip-irrigated rice by improving photosynthetic performance and enhancing nitrogen metabolism through optimizing water and nitrogen management

**DOI:** 10.3389/fpls.2023.1075625

**Published:** 2023-02-23

**Authors:** Lei Zhao, Qingyun Tang, Zhiwen Song, Yongan Yin, Guodong Wang, Yuxiang Li

**Affiliations:** ^1^ Key Laboratory of Oasis Eco-Agriculture, Xinjiang Production and Construction Group, Shihezi University, Shihezi, Xinjiang, China; ^2^ Xinjiang Tianye Group Ltd., Shihezi, Xinjiang, China; ^3^ Institute of Farmland Water Conservancy and Soil-Fertilizer, Xinjiang Academy of Agricultural Reclamation Science or Key Laboratory of Northwest Oasis Water-Saving Agriculture, Ministry of Agriculture and Rural Affairs, Shihezi, Xinjiang, China

**Keywords:** drip-irrigated rice, water and nitrogen management, grain yield, nitrogen metabolism, photosynthesis

## Abstract

Drip irrigation under plastic film mulching is an important technique to achieve water-conserving and high-efficiency rice (*Oryza sativa* L.) production in arid areas, but the grain yield of drip-irrigated rice is much lower than the expected yield (10.9-12.05 t·hm^-2^) in practical production applications. Therefore, we hope to further understand the photosynthetic physiological mechanism of drip-irrigated rice yield formation by optimizing water and nitrogen management during the growth period and provide a scientific reference for improving yield and nitrogen use efficiency (NUE) of drip-irrigated rice in arid areas. In 2020 and 2021, T-43 (a drought-resistant; V1) and Liangxiang-3 (a drought-sensitive cultivar; V2) were cultivated under two water treatments (W_1_: limited drip irrigation, 10200 m^3^·hm^-2^; W_2_: deficit drip irrigation, 8670 m^3^·hm^-2^) and three nitrogen fertilization modes with different ratios of seedling fertilizer:tillering fertilizer:panicle fertilizer:grain fertilizer (N_1_, 30%:50%:13%:7%; N_2_, 20%:40%:30%:10%; and N_3_, 10%:30%:40%:20%). The photosynthetic characteristics, nitrogen metabolism, yield, and NUE were analysed. The results showed that compared with other treatments, the W_1_N_2_ resulted in 153.4-930.3% higher glutamate dehydrogenase (GDH) contents and 19.2-49.7% higher net photosynthetic rates (*P*_n_) in the leaves of the two cultivars at 20 days after heading, as well as higher yields and NUE. The two cultivars showed no significant difference in the physiological changes at the panicle initiation stage, but the *P*_n_, abscisic acid (ABA), indole acetic acid (IAA), gibberellic acid (GA_3_), and zeatin riboside (ZR) levels of V1 were higher than those of V2 by 53.1, 25.1, 21.1, 46.3 and 36.8%, respectively, at 20 days after heading. Hence, V1 had a higher yield and NUE than V2. Principal component analysis revealed that *P*_n_ and GDH were the most important physiological factors affecting rice yield performance. In summary, the W_1_N_2_ treatment simultaneously improved the yield and NUE of the drought-resistant rice cultivar (T-43) by enhancing the photosynthetic characteristics and nitrogen transport capacity and coordinating the balance of endogenous hormones (ABA, IAA, GA_3_, and ZR) in the leaves.

## 1 Introduction

Rice is among the major food crops worldwide, and more than half of the global population depends on rice (Patel et al., 2010). In 2020, the rice planting area in China reached 3.008×10^7^ hectares, with a yield of 211.86 million tons, accounting for 37.3% of global rice production (National Bureau of Statistics of China, 2020). The traditional flooding irrigation rice cultivation system has the problems of high water consumption and low water use efficiency (WUE), so the development of a water-saving rice production system has become an urgent task (Monaco and Sali, 2018). Drip irrigation rice cultivation under mulch is the most effective technology to improve the efficiency of rice irrigation water in arid areas. Compared with traditional flooding irrigation, drip-irrigated rice can reduce ineffective evaporation between plants, surface runoff and underground leakage and increase the root length density, root activity and aboveground dry matter accumulation (He et al., 2016). It has the potential to achieve high rice yield (12.1 t·hm^-2^), water savings (65%), fertilizer savings (20%) and cost savings (17.2% cost reduction) (Chen et al., 2013). In the actual production of rice in arid areas, drip-irrigated rice significantly improves WUE (1.08-1.13 kg·m^-3^) (Hang et al., 2022), but the grain yield (5.9-8.7 t·hm^-2^) is much lower than the expected target (10.9-12.05 t·hm^-2^) (Chen et al., 2013; Hang et al., 2022).

Meseret et al. (2022) concluded that split applications of nitrogen fertilizer according to the demand of the crops and at the key growth stages of crops have an important effect on crop growth and yield. Previous studies have shown that the appropriate postponement of nitrogen fertilization for rice is beneficial to forming good root morphology (Yan et al., 2015) while increasing the tillering rate (Zhang et al., 2011), the activity of nitrogen metabolism enzymes and the photosynthetic rate (Zhang et al., 2016), and the dry matter accumulation in the aboveground parts (Yang et al., 2015), thereby improving grain yield and NUE while reducing the total greenhouse gas emissions and emission intensity (Zhang et al., 2022a). Sun et al. (2017) also showed that appropriate increase panicle fertilizer was conducive to the transport of nutrients to grains and to maintaining an effective leaf area rate (proportion of the leaf area of the three leaves above the effective stem in the population leaf area) and high zeatin and zeatin riboside (Z+ZR) contents in roots after heading. However, the physiological processes of the effect of timed application of nitrogen fertilizer on rice yield formation under drip irrigation remain unclear. Therefore, we propose that the WUE and fertilizer use efficiency (NUE) can be simultaneously improved by optimizing the water and fertilizer supply during the rice growth period and fully tapping the water-saving and high-yield potential of this crop by harnessing the advantages of precision irrigation and fertilization of drip irrigation technology.

At present, water and fertilizer management during drip irrigation for rice production is primarily based on the water and fertilizer management modes of flooding irrigation, which results in increasingly prominent problems of low WUE, NUE, resource waste, and chemical fertilizer pollution. Therefore, studying the water and nitrogen supply modes suitable for drip irrigation for rice cultivation in arid areas and understanding the photosynthetic physiological mechanism of water nitrogen supply affecting drip-irrigated rice yield formation are of great importance to the further exploration of biological water savings in rice crops. In our previous study, reducing the irrigation amount (from 12000 m^3^·hm^-2^ to 10200 m^3^·hm^-2^) increased the WUE without significantly reducing the yield of drought-resistant rice varieties in drip irrigation under plastic film mulching (Zhao et al., 2023). On this basis, the effects of further optimizing the water and nitrogen management modes on the photosynthetic physiological characteristics and yield formation of drip-irrigated rice were investigated to screen suitable water and nitrogen supply modes for improving the yield. The physiological mechanisms affecting yield formation were also elucidated from the physiological perspectives of nitrogen metabolism enzymes, photosynthesis, fluorescence, antioxidant enzymes, hormones. This study is expected to provide a scientific reference for improving the yield and NUE of drip-irrigated rice in arid areas.

## 2 Materials and methods

### 2.1 Test area

**Figure 1:**
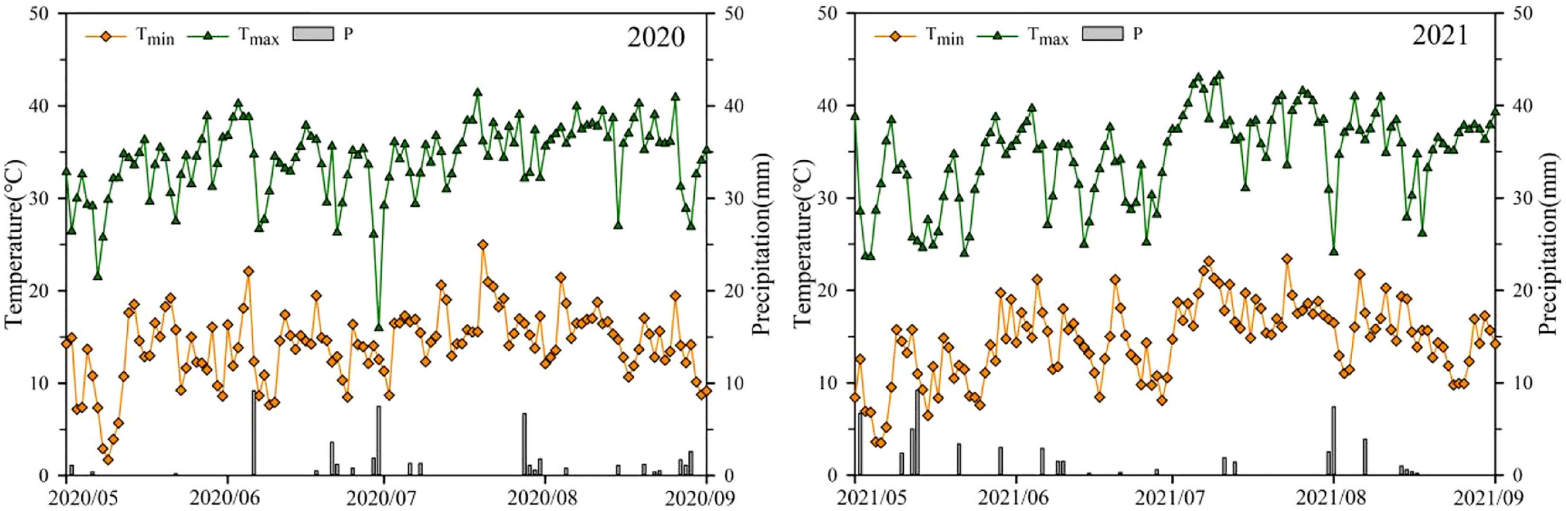
Temperature and precipitation in the test area during the rice growth periods of 2020 and 2021.

This experiment was conducted at the experimental field of the Xinjiang Academy of Agricultural and Reclamation Sciences (longitude 86°03’E, latitude 44°19’N) from 2020 to 2021. The test area has an arid to semiarid continental climate. The average temperature during the growth period of rice was 17.7°C and 18.7°C, and the average rainfall was 0.3 mm and 0.4 mm in 2020 and 2021, respectively. Changes in precipitation and temperature during the rice growth period (May 1 to September 30) are shown in **Figure 1**. The experimental soil was loam in texture with an organic matter content of 11.21 g·kg^-1^, total N of 0.74 g·kg^-1^, alkaline N of 0.61 g·kg^-1^, available P of 0.51 g·kg^-1^, and available K of 93 g·kg^-1^.

### 2.2 Experimental design

A three-factor randomized block design was used in this experiment. Each treatment had a plot dimension of 22.99 m^2^ (6.13 m×3.75 m), with three replicates for each treatment. The tested rice cultivars, T-43 (V1, a drought-resistant high-yield variety) and Liangxiang-3 (V2, a drought-sensitive variety), are frequently planted in Xinjiang. The seeds of T-43 and Liangxiang-3 were provided by the Institute of Agricultural Science, Tianye Company (Xinjiang, China). The tested rice cultivars were cultivated with two water treatments (W_1_, limited drip irrigation at 10,200 m^3^·hm^-2^, which was 85% of the current drip irrigation rate commonly used in rice production in Xinjiang based on preliminary experimental results and W_2_, deficit drip irrigation at 8670 m^3^·hm^-2^, which was 85% of the drip irrigation rate used in W_1_ during the entire growth period) and three nitrogen fertilization modes with the same total nitrogen application amounts (pure N: 300 kg·hm^-2^) and different ratios of seedling fertilizer:tillering fertilizer:panicle fertilizer:grain fertilizer (N_1_: 30%:50%:13%:7%; N_2_: 20%:40%:30%:10%; and N_3_: 10%:30%:40%:20%) (see **Table 1** for details). P_2_O_5_ and K_2_O fertilizer were applied with water, and the application rates were 150 kg·hm^-2^ and 135 kg·hm^-2^, respectively. Fifty percent of the applied P_2_O_5_ and K_2_O was used as seedling fertilizer, and the other 50% was used as panicle fertilizer. Urea (N, 46%) and potassium dihydrogen phosphate (P_2_O_5_, 52.1%; K_2_O, 34.6%) were used as the nitrogen fertilizer and the P_2_O_5_ and K_2_O fertilizer, respectively.

**Table 1:** N rate for drip irrigation rice under plastic film mulching.

Treatments	Irrigation amount (m^3^·hm^-2^)	Nitrogen fertilization amount (kg·hm^-2^)	Seedling: Tiller: Spike: Grain nitrogen	N fertilizer rate (kg·hm^-2^)							
				Seedlings nitrogen		Tiller nitrogen			Spike nitrogen		Grain nitrogen
				20 days after sowing	28 days after sowing	36 days after sowing	45 days after sowing	54 days after sowing	78 days after sowing	88 days after sowing	95 days after sowing
W_1_N_1_	10200	300	30%:50%:13%:7%	45.0	45.0	50.0	50.0	50.0	19.5	19.5	21.0
W_1_N_2_			20%:40%:30%:10%	30.0	30.0	40.0	40.0	40.0	45.0	45.0	30.0
W_1_N_3_			10%:30%:40%:20%	15.0	15.0	30.0	30.0	30.0	60.0	60.0	60.0
W_2_N_1_	8670		30%:50%:13%:7%	45.0	45.0	50.0	50.0	50.0	19.5	19.5	21.0
W_2_N_2_			20%:40%:30%:10%	30.0	30.0	40.0	40.0	40.0	45.0	45.0	30.0
W_2_N_3_			10%:30%:40%:20%	15.0	15.0	30.0	30.0	30.0	60.0	60.0	60.0

W_1_ and W_2_ represent limited irrigation (10200 m^3^·hm^-2^) and deficit irrigation (8670 m^3^·hm^-2^), while N_1_, N_2_, and N_3_ represent different ratios of seedling fertilizer:tillering fertilizer:panicle fertilizer:grain fertilizer (30%:50%:13%:7%; 20%:40%:30%:10%; and 10%:30%:40%:20%, respectively).

**Figure 2:**
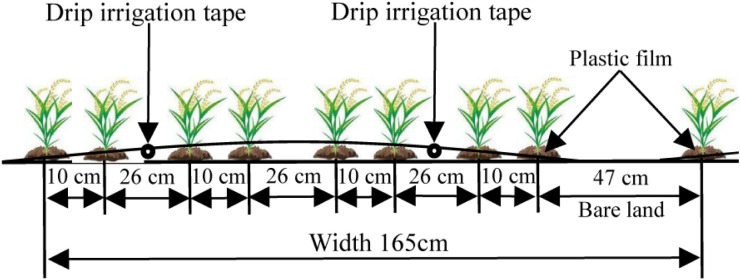
Planting mode for drip irrigation under plastic film mulching.

The experiment used a planting pattern of one film, two tubes, and eight rows. The sowing width was 1.65 m, the plant spacing was 10 cm, and the row spacing was 10 cm + 26 cm + 10 cm + 26 cm + 10 cm + 47 cm, as shown in **Figure 2**. The drip irrigation tape placement, plastic film mulching, spot seeding, and covering of the seeds with soil were all completed at one time. During the growth period, precision management was performed to control pests and weeds in a timely manner. The seeds were sown on May 1, 2020, and May 4, 2021. After being sown, the seeds were drip irrigated with water, and the seedlings were released from the plastic film after emergence. A total of 6 to 8 seedlings were preserved in each planting hole, and the rice plants were harvested on September 30 in both years. The irrigation frequency was once every 3 days before and once every 2 days after jointing, and irrigation was stopped until 15 days before maturity. The drip irrigation tape used in this study was single-wing labyrinth drip irrigation tape produced by Xinjiang Tianye Co., Ltd. (Xinjiang, China) with an emitter spacing of 30 cm and a flow rate of 1.8 L·h^-1^. The plots were spaced 1 m apart to prevent lateral water seepage between plots. A water meter (measurement accuracy, 0.001 m^3^) was used to measure the amount of irrigation water applied through the drip irrigation system. Other field management measures were performed using drip irrigation methods commonly used under plastic film mulching in high-yielding rice fields.

### 2.3 Measurement methods

#### 2.3.1 Determination of gas exchange parameters

The gas exchange parameters were measured at 11:00-13:00 at the panicle initiation (June 25 and June 30 in 2020 and 2021, respectively) and heading stages (July 11 and July 16 in 2020 and 2021, respectively) and 20 days after heading (July 31 and August 16 in 2020 and 2021, respectively). The size of the air chamber gasket was 2×3 cm^2^. A 2 × 3 light source provided independent control of red and blue light intensities (6400-02B). The photosynthetically active radiation, CO_2_ concentration in the leaf chamber, and leaf temperature were set at 1800 µmol m^-2^·s^-1^, 400 µmol·mol^-1^, and 30 ± 4°C, respectively. Healthy, fully extended flag leaves (the first fully expanded leaf under the heart leaf before heading) were labelled with tags. Three planting hills were selected for each treatment, and five flag leaves were selected from each planting hill. The net photosynthetic rate (*P*_n_), stomatal conductance (*G*_s_), transpiration rate (*T*_r_), and intercellular CO_2_ concentration (*C*_i_) were measured using a portable photosynthesis system (LI-6400XT, LI-COR, USA).

#### 2.3.2 Determination of chlorophyll fluorescence parameters

After the photosynthetic parameters were measured, the chlorophyll fluorescence parameters of the labelled leaves were measured using a portable fluorometer (PAM-2500, Heinz Walz GmbH, Germany) at the panicle initiation and heading stage and 20 days after the heading stage. After the leaves were dark-adapted for 30 min using dark-adapted leaf clips (DLC-B), the measurement light was turned on, and the dark-adapted initial fluorescence (*F*_o_) and maximum fluorescence (*F*_m_) were measured to obtain the maximum quantum efficiency (*F*_v_/*F*_m_) of the dark-adapted photosystem II (PSII). Then, the photochemical light was turned on (PAR=617 μmol·m^-2^·s^-1^), and the actual photochemical quantum efficiency (*Y*(II)), photochemical quenching coefficient (q*P*), and non-photochemical quenching coefficient (q*N*) of the leaves were measured under the corresponding light intensity, with three replicates for each treatment. The calculation formulas are defined as follows:


(1) $$F_{\text{y}}/F_{\text{m}}=(F_{\text{m}}-F_{\text{o}})/F_{\text{m}}$$

(2) Y(II)=(Fm'−Ft)/Fm'

(3) qP=(Fm'−Ft)/(Fm'−Fo)

(4) qN=(Fm−Fm')/(Fm−Fo)

where *F*_v_/*F*_m_, *Y*(II), q*P*, and q*N* are the maximal photochemical efficiency of PSII, actual photochemical quantum efficiency, photochemical quenching, and non-photochemical quenching of the leaf, respectively, and *F*_m_’, *F*_t_, *F*_o_, and *F*_m_ are the maximum fluorescence yield, actual fluorescence intensity at any time, initial fluorescence, and maximum fluorescence yield, respectively, when the PSII reaction centres are all in the off state at saturation pulses under light.

#### 2.3.3 Determining the activities of antioxidant enzymes and nitrogen metabolism enzymes

Rice plants with consistent growth and development (sampling by population mean stem number) were selected at the panicle initiation and heading stage and 20 days after heading. The flag leaves (the first fully expanded leaf under the heart leaf before heading) were sampled and frozen in liquid nitrogen and stored at -80°C to analyse their nitrogen metabolic enzymes and antioxidant enzymes. Nitrate reductase (NR) activity was determined according to the *in vitro* method described by Li et al. (2000). The enzyme activity was expressed in the number of micrograms of NaNO_2_ produced per gram of sample per hour (μg/(h·g) (calculated as NaNO_2_, the same below). Glutamine synthetase (GS) was determined according to the method described by Wang et al. (2005). Glutamate dehydrogenase (GDH) was determined according to the method described by Masclaux et al. (2000). The superoxide dismutase (SOD) activity and peroxidase (POD) activity were measured according to the method described by Qiu et al. (2010). Catalase (CAT) activity was measured using the UV absorption method (Zeng et al., 1991).

#### 2.3.4 Hormone determination

Rice plants with consistent growth and development (sampling by population mean stem number) were selected at the panicle initiation and heading stage and 20 days after heading. The flag leaves (the first fully expanded leaf under the heart leaf before heading) were frozen in liquid nitrogen. The contents of abscisic acid (ABA), indole acetic acid (IAA), gibberellic acid (GA_3_), and zeatin riboside (ZR) were determined by enzyme-linked immunosorbent assay (ELISA) (Onoda et al., 2017), with three replicates for each treatment.

#### 2.3.5 Yield and NUE

The number of effective panicles, the 1000-grain weight, the total number of grains per panicle, the number of filled grains per panicle, and the seed setting rate were analysed at the rice maturity stage. The yield was calculated based on a moisture content of 14.5%. Three planting hills were selected for each treatment, with three replicates for each treatment. The formula for calculating the NUE is given as follows:


(5) PFP=Y/N×100%

where PFP is the partial factor productivity (kg·kg^-1^), Y is the yield (t·hm^-2^), and N is the nitrogen application rate (kg·hm^-2^).

### 2.4 Data analysis and statistical methods

The data were subjected to analysis of variance (ANOVA) using SPSS 26.0 software (SPSS, Chicago, IL, USA), and differences among treatments were tested according to Duncan’s multiple range test at a 5% level of significance. Cultivar, water, nitrogen and their interactions were treated as fixed factors, and replication was considered a random factor. SigmaPlot 14.0 software (Systat Software Inc., San Jose, CA, USA) was used to present the data in the figures. Principal component analysis of impact factors was performed using the “FactoExtra” package in R 4.0.5 software.

## 3 Results

### 3.1 Effects of water and nitrogen management on the activities of nitrogen metabolism enzymes

**Figure 3:**
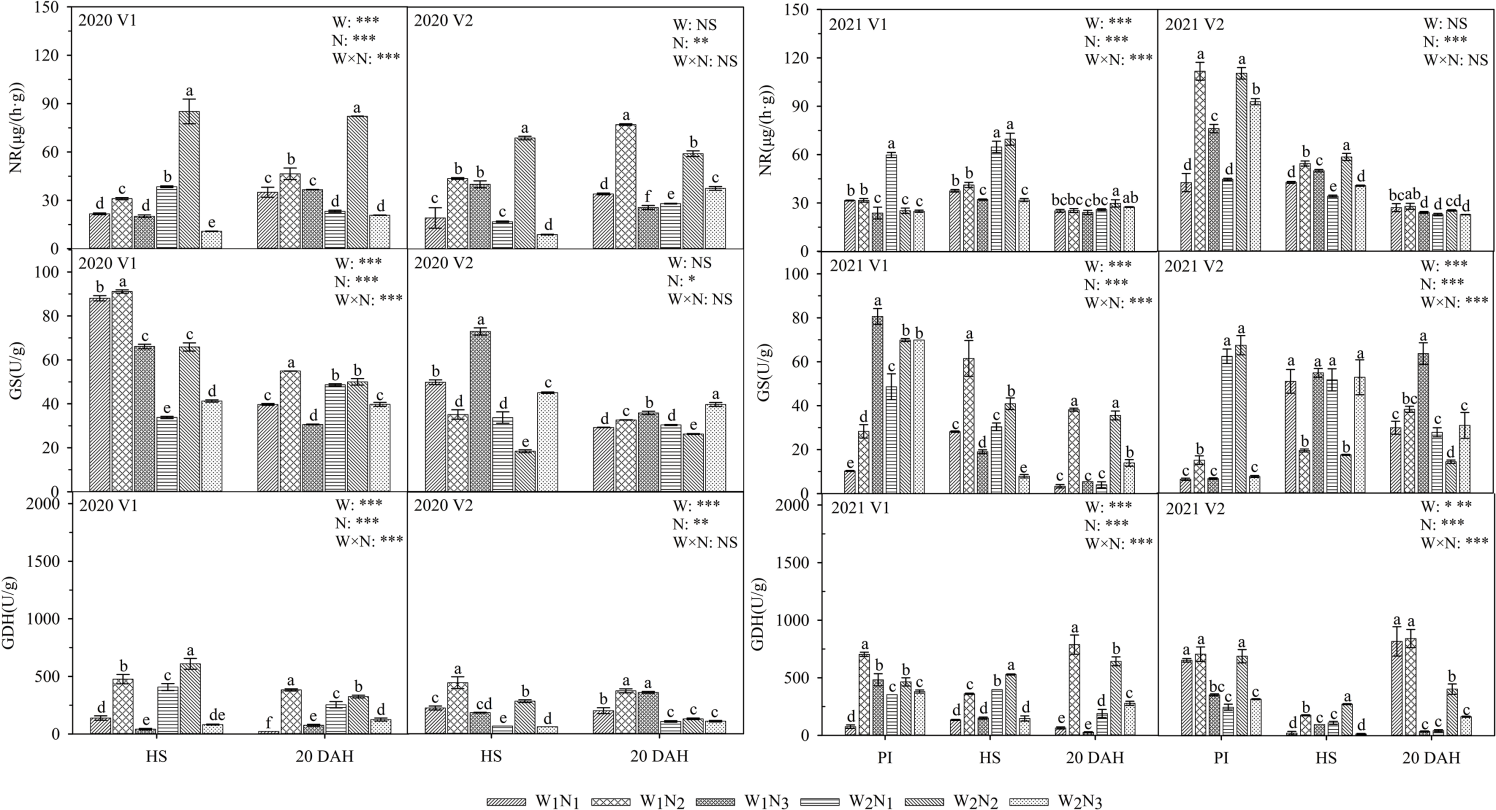
Effects of different water and nitrogen treatments on N metabolism enzyme activities in rice leaves at different growth stages. The data are presented as the means of three replicates (n = 3). Error bars indicate standard error (SE). W_1_ and W_2_ represent limited irrigation (10200 m^3^·hm^-2^) and deficit irrigation (8670 m^3^·hm^-2^), while N_1_, N_2_, and N_3_ represent different ratios of seedling fertilizer:tillering fertilizer:panicle fertilizer:grain fertilizer (30%:50%:13%:7%; 20%:40%:30%:10%; and 10%:30%:40%:20%, respectively). PI, HS, and 20 DAH represent panicle initiation, heading stages, and 20 days after heading, respectively. V1 and V2 represent T-43 and Liangxiang-3, respectively. * represents a significant difference at the 0.05 level; ** represents a significant difference at the 0.01 level; *** represents a significant difference at the 0.001 level.

As shown in **Figure 3**, the interaction of water and nitrogen had a significant effect on the NR, GS, and GDH of V1, and nitrogen application had a significant effect on the NR, GS, and GDH of V2. At the panicle initiation stage, the GDH in V1 under W_1_ increased first and then decreased, reaching a maximum under N_2_, and GS in V1 reached a maximum under N_3_; the GS and GDH in V2 under W_2_ increased first and then decreased with the postponement of nitrogen fertilization. From the heading stage to 20 days after heading, the NR, GS, and GDH in V1 under W_1_ and W_2_ all increased first and then decreased with the postponement of nitrogen fertilization and reached a maximum at N_2_; NR and GDH in V2 had the same trends as those in V2, whereas GS in V2 reached a maximum at N_3_. The GS in V1 under W_1_N_2_ was 26.5-301.7% higher than that under the other treatments from heading to 20 days after heading, and the GDH in V1 and V2 under W_1_N_2_ was 44.6-1131.9% higher than that under the other treatments at 20 days after heading. The GS content under the W_1_N_2_ treatment was 8.6-96.1% higher than that under the W_2_N_2_ treatment from heading to 20 days after heading, but the NR content was lower than that under the W_2_N_2_ treatment at the heading stage. A comparison of the two varieties showed that the GDH and GS in 2020 of V1 under the W_2_N_2_ treatment were higher than those of V2 at 20 days after heading.

### 3.2 Effects of water and nitrogen management on photosynthetic capacity

**Figure 4:**
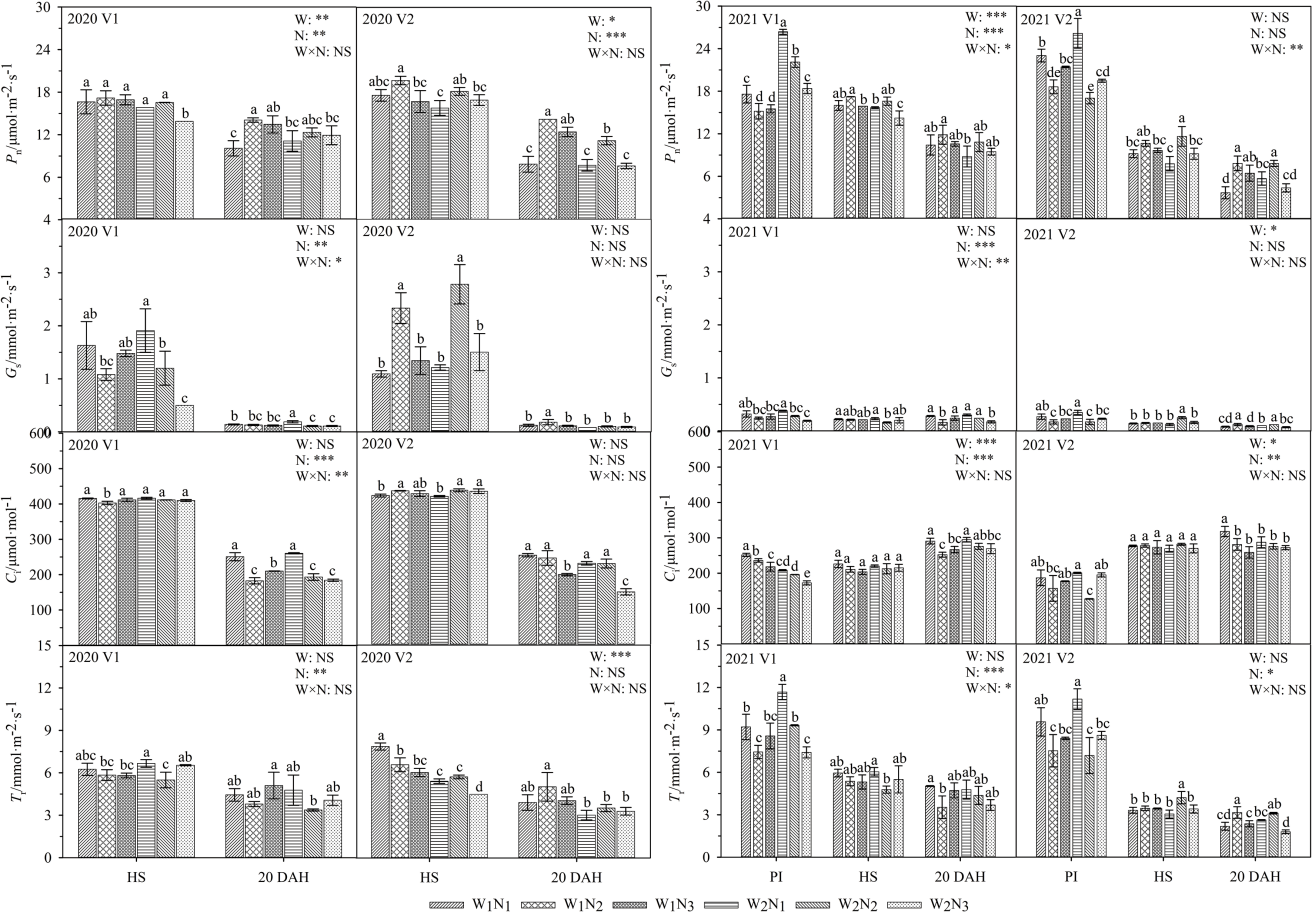
Effects of different water and nitrogen treatments on photosynthetic parameters in rice leaves at different growth stages. The data are presented as the means of three replicates (n = 3). Error bars indicate standard error (SE). W_1_ and W_2_ represent limited irrigation (10200 m^3^·hm^-2^) and deficit irrigation (8670 m^3^·hm^-2^), while N_1_, N_2_, and N_3_ represent different ratios of seedling fertilizer:tillering fertilizer:panicle fertilizer:grain fertilizer (30%:50%:13%:7%; 20%:40%:30%:10%; and 10%:30%:40%:20%, respectively). PI, HS, and 20 DAH represent panicle initiation, heading stages, and 20 days after heading, respectively. V1 and V2 represent T-43 and Liangxiang-3, respectively. * represents a significant difference at the 0.05 level; ** represents a significant difference at the 0.01 level; *** represents a significant difference at the 0.001 level.

As shown in **Figure 4**, the interaction of water and nitrogen had a significant effect on the *G*_s_ of V1. From the heading stage to 20 days after heading, the *P*_n_ of the two cultivars under W_1_ and W_2_ increased first and then decreased and reached a maximum at N_2_; the *G*_s_, *C*_i_, and *T*_r_ of V2 showed similar trends relative to the *P*_n_, whereas the *G*_s_, *C*_i_, and *T*_r_ in V1 decreased significantly. The *P*_n_ of V1 under W_1_N_2_ was 6.7-23.2% higher and that of V2 was 0.5-64.6% higher than that of the other treatments from heading to 20 days after heading, but the difference was not significant. Compared with that under W_1_, the *P*_n_ under the N_2_ treatment was 3.8-13.4% higher from heading to 20 days after heading. A comparison of the two cultivars revealed that the *P*_n_ of V1 was 19.6-103.6% higher than that of V2 at 20 days after heading.

### 3.3 Effect of water and nitrogen management on chlorophyll fluorescence parameters

**Figure 5:**
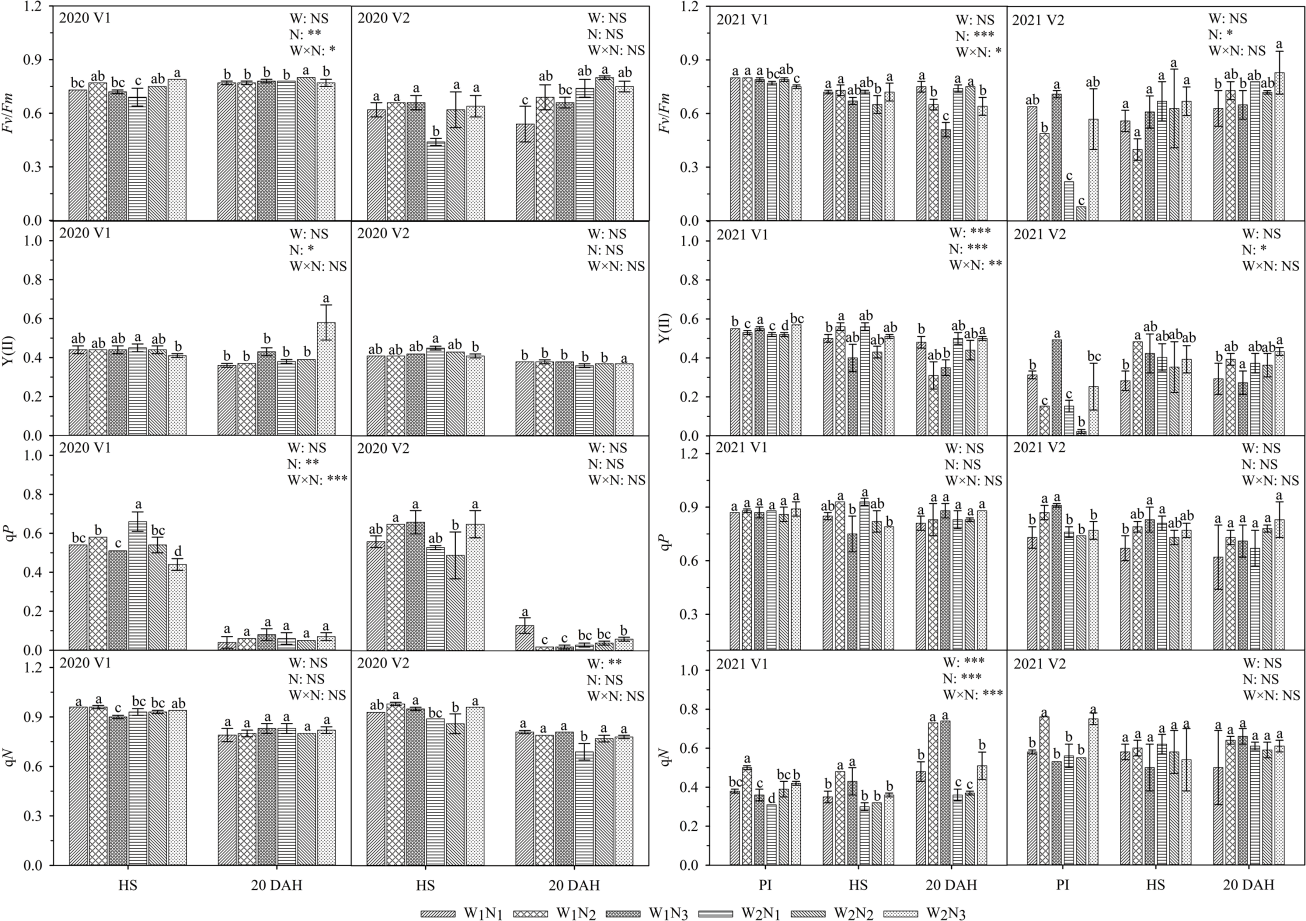
Effects of different water and nitrogen treatments on chlorophyll fluorescence in rice leaves at different growth stages. The data are presented as the means of three replicates (n = 3). Error bars indicate standard error (SE). W_1_ and W_2_ represent limited irrigation (10200 m^3^·hm^-2^) and deficit irrigation (8670 m^3^·hm^-2^), while N_1_, N_2_, and N_3_ represent different ratios of seedling fertilizer:tillering fertilizer:panicle fertilizer:grain fertilizer (30%:50%:13%:7%; 20%:40%:30%:10%; and 10%:30%:40%:20%, respectively). PI, HS, and 20 DAH represent panicle initiation, heading stages, and 20 days after heading, respectively. V1 and V2 represent T-43 and Liangxiang-3, respectively. * represents a significant difference at the 0.05 level; ** represents a significant difference at the 0.01 level; *** represents a significant difference at the 0.001 level.

As shown in **Figure 5**, the interaction of water and nitrogen had a significant effect on the *F*_v_/*F*_m_ of V1. At the panicle initiation stage, the q*P* and q*N* of V1 under W_1_ increased first and then decreased. At the heading stage, the *F*_v_/*F*_m_ and *Y*(II) of V1 and the q*N* of V2 under W_1_ increased first and then decreased. At 20 days after heading, the q*N* of V1 and the *F*_v_/*F*_m_ and *Y*(II) of V2 under W_1_ and W_2_ gradually increased. A comparison of the two cultivars showed that the *F*_v_/*F*_m_ of V1 was 9.1-50.4% higher than that of V2 at the heading stage and that the q*P* of V1 was 4.0-130.7% higher than that of V2 at 20 days after heading (except for W_1_N_1_).

### 3.4 Effects of water and nitrogen management on hormone contents

**Figure 6:**
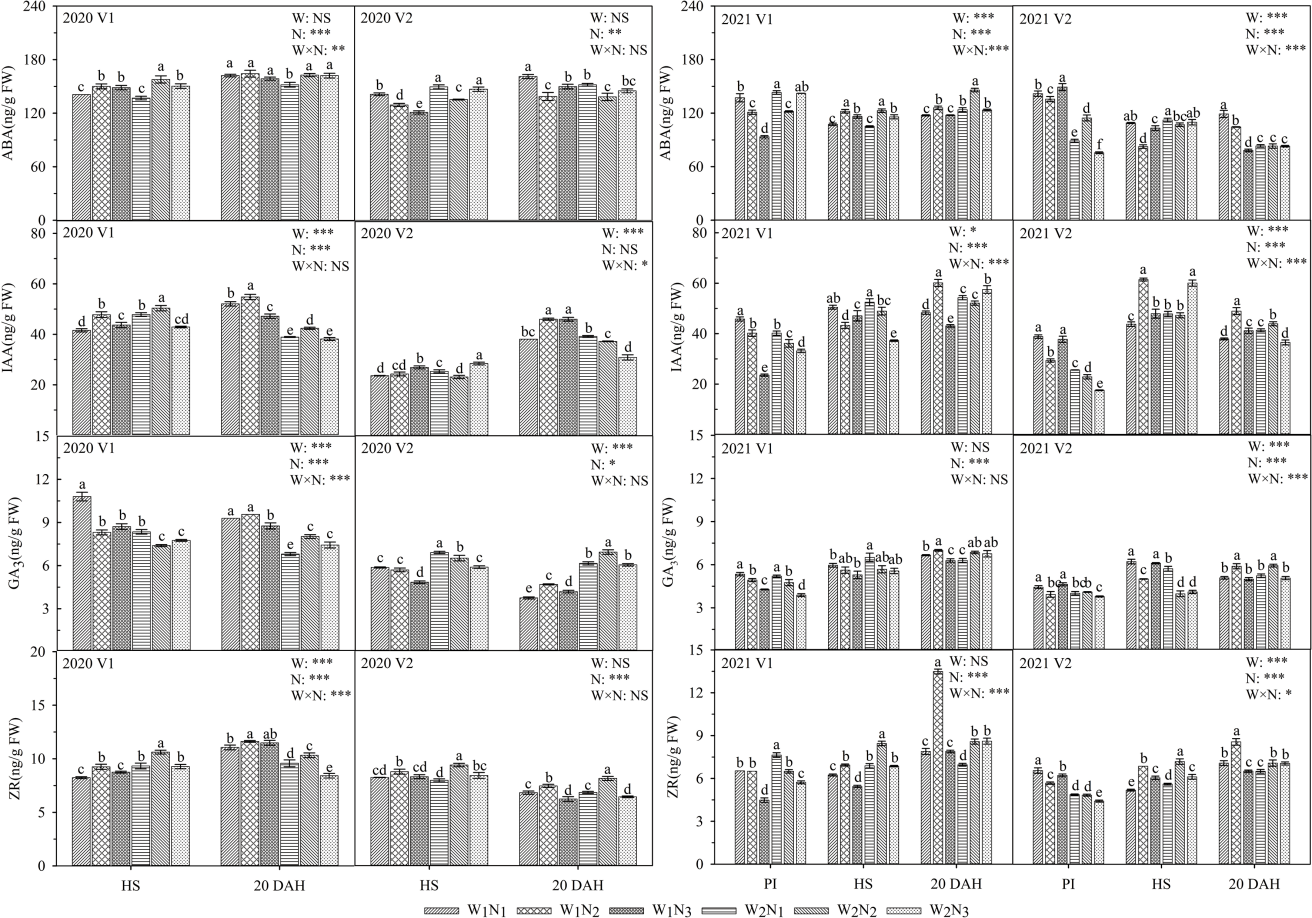
Effects of different water and nitrogen treatments on endogenous hormones in rice leaves at different growth stages. The data are presented as the means of three replicates (n = 3). Error bars indicate standard error (SE). W_1_ and W_2_ represent limited irrigation (10200 m^3^·hm^-2^) and deficit irrigation (8670 m^3^·hm^-2^), while N_1_, N_2_, and N_3_ represent different ratios of seedling fertilizer:tillering fertilizer:panicle fertilizer:grain fertilizer (30%:50%:13%:7%; 20%:40%:30%:10%; and 10%:30%:40%:20%, respectively). PI, HS, and 20 DAH represent panicle initiation, heading stages, and 20 days after heading, respectively. V1 and V2 represent T-43 and Liangxiang-3, respectively. * represents a significant difference at the 0.05 level; ** represents a significant difference at the 0.01 level; *** represents a significant difference at the 0.001 level.

As shown in **Figure 6**, the interaction of water and nitrogen had a significant effect on the ABA and ZR of V1 and had a significant effect on the IAA of V2. At the panicle initiation stage, the ABA, IAA, GA_3_, and ZR activities of V1 under W_1_ gradually decreased with the postponement of nitrogen fertilization, and the ABA, IAA, GA_3_, and ZR activities of V2 decreased first and then increased. At the heading stage, the IAA in V2 under W_2_ reached a maximum at N_3_. At 20 days after heading, the IAA and GA_3_ in V1 under W_1_ and W_2_ decreased first and then increased, and the trend of GA_3_ in V2 was consistent with that in V1. The IAA, GA_3_, and ZR of V1 under the W_1_N_2_ treatment at 20 days after heading were 14.8-27.9, 4.0-25.9, and 34.8-57.5% higher than those of the other treatments, respectively, and notably, the IAA of V1 was significantly higher than those of the other treatments (*P* ≤ 0.05). The IAA content of the two cultivars under the W_1_N_2_ treatment was 11.4-29.3% higher than that under W_2_N_2_ at 20 days after heading. A comparison of the two cultivars showed that the ABA content of V1 was 6.6-88.9% higher than that of V2 under W_2_ and was 3.2-59.4% lower than that of V2 under W_1_ at the panicle initiation stage. The ZR and GA_3_ of V1 under W_2_ were 11.1-20.1 and 17.4-33.6% higher than those of V2, respectively, at the heading stage. The ABA, IAA, GA_3_, and ZR of V1 were 0.8-46.8, 15.6-89.1, 20.0-45.1, and 3.2-39.9% higher than those of V2, respectively, at 20 days after heading.

### 3.5 Effects of water and nitrogen management on antioxidant enzyme activities

**Figure 7:**
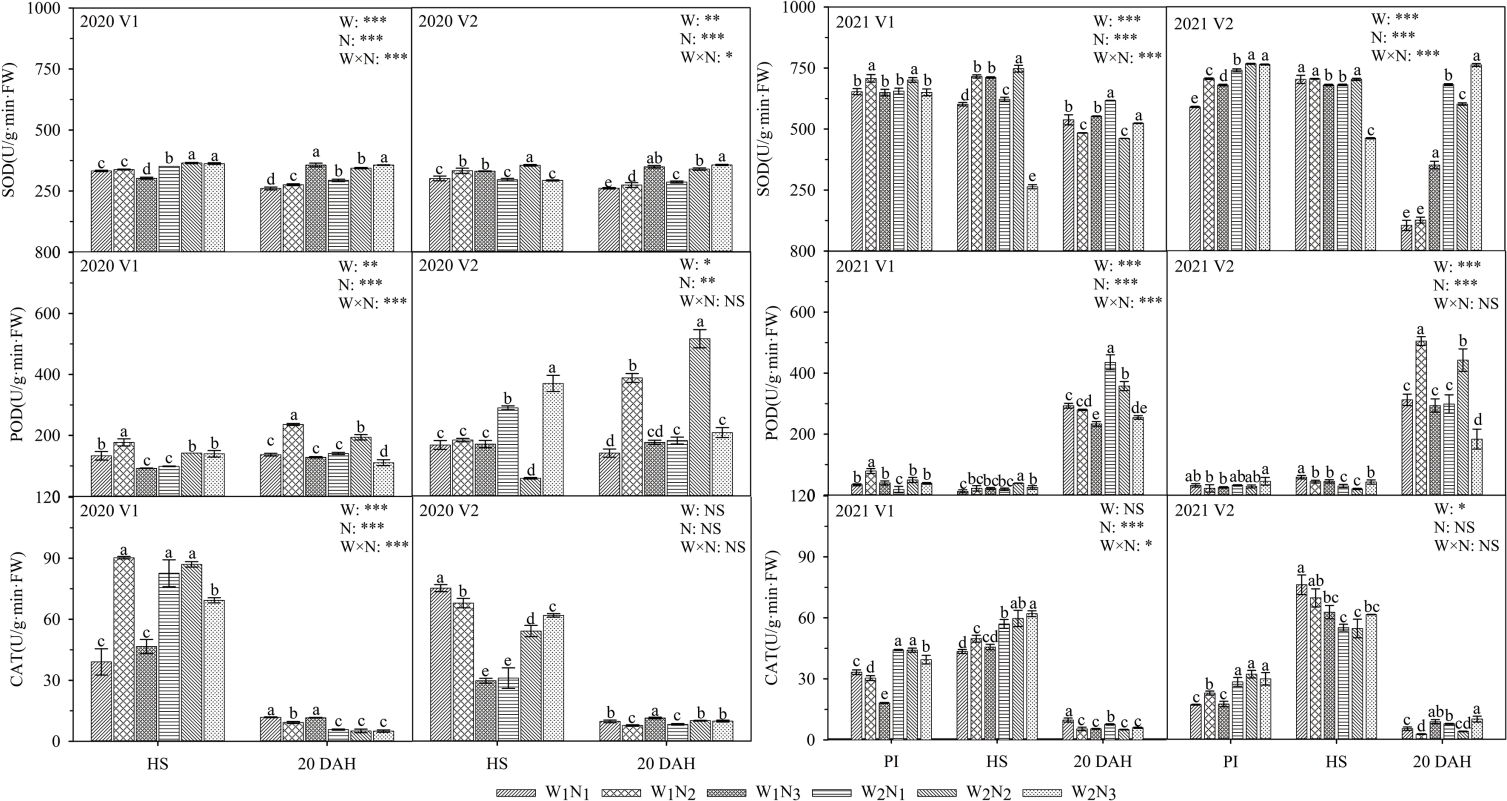
Effects of different water and nitrogen treatments on antioxidative enzyme activities in rice leaves at different growth stages. The data are presented as the means of three replicates (n = 3). Error bars indicate standard error (SE). W_1_ and W_2_ represent limited irrigation (10200 m^3^·hm^-2^) and deficit irrigation (8670 m^3^·hm^-2^), while N_1_, N_2_, and N_3_ represent different ratios of seedling fertilizer:tillering fertilizer:panicle fertilizer:grain fertilizer (30%:50%:13%:7%; 20%:40%:30%:10%; and 10%:30%:40%:20%, respectively). PI, HS, and 20 DAH represent panicle initiation, heading stages, and 20 days after heading, respectively. V1 and V2 represent T-43 and Liangxiang-3, respectively. * represents a significant difference at the 0.05 level; ** represents a significant difference at the 0.01 level; *** represents a significant difference at the 0.001 level.

As shown in **Figure 7**, the interaction of water and nitrogen had a significant effect on the SOD and POD of V1, and nitrogen application had a significant effect on the SOD of V2. The POD activity gradually increased with plant growth, while the CAT activity decreased. At the panicle initiation stage, the SOD activity of the two cultivars first increased and then decreased with the postponement of nitrogen fertilization. At the heading stage, the CAT activity of V1 under W_1_ first increased and then decreased with the postponement of nitrogen fertilization, while the CAT activity of V2 reached a maximum under N_1_; the CAT activity of V2 under W_2_ reached a maximum under N_3_. The comparison of the two cultivars showed that the SOD activity of V1 under N_2_ was 3.2% higher than that of V2 at the heading stage, and the SOD activities of V1 under W_1_ were 29.8-207.3% higher than those of V2 at 20 days after heading. The POD of V2 under W_1_ was 4.7-72.4% higher than that of V1 at 20 days after heading.

### 3.6 Effect of water and nitrogen management on rice yield and NUE

**Figure 8:**
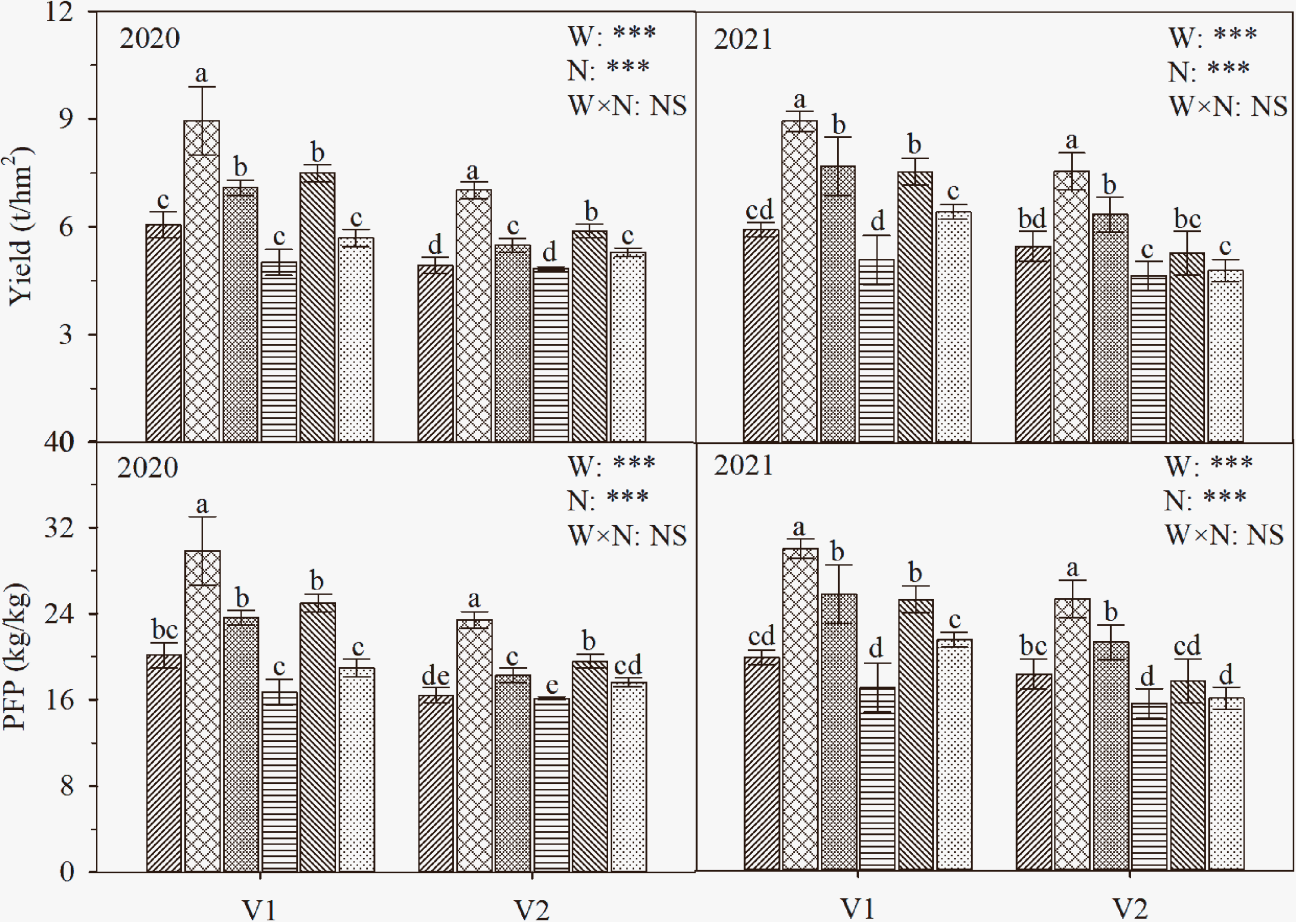
The yield and PFP of rice under different treatments. The data are presented as the means of three replicates (n = 3). Error bars indicate standard error (SE). W_1_ and W_2_ represent limited irrigation (10200 m^3^·hm^-2^) and deficit irrigation (8670 m^3^·hm^-2^), while N_1_, N_2_, and N_3_ represent different ratios of seedling fertilizer:tillering fertilizer:panicle fertilizer:grain fertilizer (30%:50%:13%:7%; 20%:40%:30%:10%; and 10%:30%:40%:20%, respectively). PI, HS, and 20 DAH represent panicle initiation, heading stages, and 20 days after heading, respectively. V1 and V2 represent T-43 and Liangxiang-3, respectively. *** represents a significant difference at the 0.001 level.

As shown in **Figure 8**, the interaction of water and nitrogen had significant effects on the yield and PFP of the two cultivars. The grain yield and PFP of the two cultivars under different water treatments first increased and then decreased with the postponement of nitrogen fertilization and reached a maximum at N_2_. Specifically, the grain yield of V1 under W_1_N_2_ was significantly higher than those of the other treatments by 19.1-77.3% (*P* ≤ 0.05), and the grain yield of V2 under W_1_N_2_ was higher than those of the other treatments by 23.4-53.8%. The trend of PFP in the two cultivars was consistent with that of yield. The comparison of the two cultivars showed that the grain yields of V1 were both 19.66% higher than those of V2 (except for W_2_N_3_ treatment of T-43 in 2020), and PFP was consistent with the trend of grain yield.

### 3.7 Correlation analysis

A principal component analysis (PCA) was performed to examine the effects of functional leaf hormones, photosynthesis, nitrogen metabolism enzymes, and antioxidant enzyme activities on rice yield and NUE. The PCA revealed that the 14 parameters were divided into PC1 (27.0% for V1 and 24.4% for V2) and PC2 (20.9% for V1 and 20.4% for V2). The yield was mainly affected by GDH and *P*_n_. The yield and NUE of V1 were closely related to *P*_n_, GS, and GDH, and those of V2 were closely related to *P*_n_, *G*_s_, GDH, and ABA. These findings further highlight the internal relationships among the key leaf gas exchange parameters, nitrogen metabolism, and grain yield of rice.

## 4 Discussion

### 4.1 Appropriate water and nitrogen management increases the yield and NUE

Studies have shown that under conventional flooding irrigation conditions, when the mean irrigation rate was 30,000 m^3^·hm^-2^ and the nitrogen application rate was 300 kg·hm^-2^, the yield was 5.9 t·hm^-2^, and the WUE and NUE were 0.2 kg·m^-3^ and 19.7 kg·kg^-1^, respectively (Wang et al., 2017). Under alternate dry−wet irrigation at the mean irrigation rate of 10416.5 m^3^·hm^-2^ and the nitrogen application rate of 270 kg·hm^-2^, the yield was 9.7 t·hm^-2^, and the WUE and NUE were 0.9 kg·m^-3^ and 36 kg·kg^-1^, respectively (Cao et al., 2021). Under drip irrigation and plastic film mulching at the mean irrigation rate of 12000 m^3^·hm^-2^ and the nitrogen application rate of 300 kg·hm^-2^, the yield was 4.9 t·hm^-2^, and the WUE and NUE were 0.4 kg·m^-3^ and 16.2 kg·kg^-1^, respectively (Wang et al., 2017). In the present study, the yield and NUE of V1 under W_1_N_2_ were 51.7 and 82.7% higher than those under flooding irrigation and 51.5 and 84.2% higher than those under drip irrigation, respectively, and those of V2 under W_1_N_2_ were 24.2 and 24.0% higher than those under flooding irrigation and 49.6 and 50.7% higher than those under drip irrigation, respectively. These findings show that the study of drip irrigation rice is of great value for promoting large-scale upland rice production and improving water and nitrogen utilization efficiency in arid areas. However, the yield and NUE of the two cultivars were not significantly different from those under alternate dry−wet irrigation, which may be related to the cultivars (Jehangir et al., 2022), ecological conditions (Naeem et al., 2022), soil types (Ye et al., 2019), and cultivation methods (Sandhu and Mahal, 2014).

In this study, the yield of V1 under W_1_N_2_ was 9.0 t·hm^-2^, which was 27.7% higher than the mean yield of rice in China; the yield of V2 under W_1_N_2_ was 7.3 t·hm^-2^, which was 4.0% higher than the mean yield of rice in China (National Bureau of Statistics of China, 2020) (**Figure 8**). However, when the irrigation amount was further reduced to W_2_, the yield of both varieties decreased, and the decrease in V1 reached a significant level. The results indicate that drip irrigation under plastic film mulching at an irrigation rate of 10200 m^3^·hm^-2^ and a seedling fertilizer:tillering fertilizer:panicle fertilizer:grain fertilizer ratio of 20%:40%:30%:10% is conducive to improving the yield and NUE of the drought-resistant rice cultivar (T-43), thus achieving the goal of improving the WUE, yield, and NUE of drip-irrigated rice.

### 4.2 Appropriate water and nitrogen management improves photosynthetic performance and yield

Previous research has indicated that water is an important factor affecting crop growth and regulating crop metabolic and defence systems (Guo et al., 2021). Appropriate water and management can improve the light transmittance and photosynthetic area of rice in the later growth periods, promote the accumulation and movement of dry matter, and increase the *P*_n_ (Xu et al., 2010). In this study, the *P*_n_ and IAA contents under W_1_N_2_ treatment were higher than those under W_2_N_2_ at 20 days after heading. These results indicate that appropriate water reduction does not impair photosynthesis in rice leaves but instead promotes the growth of rice 20 days after heading (Guo et al., 2021). However, too little water will produce an inhibitory effect, resulting in increased ABA at the heading stage. On the one hand, this finding implies that rice leaves have entered the senescence process at the heading stage, prompting plants to complete growth rapidly under limited water conditions (Luo et al., 2021); on the other hand, higher ABA may be a physiological mechanism to facilitate stress resistance in rice (Chen et al., 2022). Therefore, we speculate that the increase in IAA and the decrease in ABA under limited irrigation (W_1_) were related to the increase in grain yield, and rice regulated stomatal formation and opening by coordinating hormone levels (**Figures 4**, **6**), delaying leaf senescence and improving the adaptability of rice leaves to drip irrigation and grain yield.

One of the most important ways to achieve high crop yield is to regulate the photosynthetic characteristics of crops and the duration during which the leaves are green through reasonable water and nitrogen management modes (Thomas and Ougham, 2014; Simkin et al., 2019). Previous research has indicated that an appropriate nitrogen supply can increase the leaf area index and photosynthetic pigment content under water deficit conditions, thereby enhancing the photosynthetic capacity and alleviating damage to photosystem II (Wu et al., 2008; Yang et al., 2021), whereas excessive N application restricts photosynthesis under water stress (Yang et al., 2021). Nitrogen not only improves photosynthesis but also acts as a stimulating factor to regulate endogenous hormone levels and the balance between various hormones to enhance crop resistance against environmental stresses. Lü et al. (2017) showed that the ultrahigh yield of hybrid rice precisely contributed to the efficient photosynthesis ensured by low levels of *G*_s_, *C*_i_, and *T*_r_ at the late growth stage. In the present study, the N_2_ treatment increased the ABA content but decreased the *G*_s_, *C*_i_, and *T*_r_ in V1 (**Figures 4**, **6**). These findings are similar to the results of Li et al. (2015) and Zhang et al. (2017). The increase in the ABA content in the leaves of V1 with the postponement of nitrogen fertilization was primarily related to the regulation of stomatal closure, and moisture reduction leads to the synthesis of a great deal of ABA to reduce the stomatal aperture (Xing et al., 2021), which reduces transpiration while maintaining a high photosynthetic rate. In contrast, the decreased ABA content and the increased *G*_s_ and *T*_r_ in V2 resulted in aggravated water stress and limited photosynthesis. This study also showed that the low input of panicle fertilizer (N_1_) resulted in higher *G*_s_, *C*_i_, and *T*_r_ and a higher ABA content in V1 during the grain formation stage than during other treatments (N_2_, N_3_) (**Figures 4**, **6**). These findings are similar to the results of Zhang et al. (2015). Higher *G*_s_, *C*_i_, and *T*_r_ and higher ABA may cause a decrease in q*P* during the photochemical process of drought damage (**Figure 5**) and consequent water loss, premature senescence, weakening of photosynthesis in the leaves, and reduced yields.

Antioxidant enzymes play an important role in delaying leaf senescence and prolonging photosynthetic time (Procházková and Wilhelmová, 2007). In the present study, under the same water conditions, appropriate postponement of nitrogen fertilization (N_2_) increased the SOD and CAT activities in V1 at the panicle initiation stage and the POD activity in V2 at 20 days after heading (**Figure 7**). These results indicated that V1 had already experienced water stress before heading, and the appropriate postponement of nitrogen fertilization (N_2_) reduced the leaf stomatal conductance and transpiration rate and increased the antioxidant enzyme (SOD and CAT) activities, thereby delaying the senescence of functional leaves 20 days after heading. Prolonged photosynthesis time reduced the *C*_i_ and improved stress resistance (Wang et al., 2020). However, V2 did not adapt to the environment with the postponement of nitrogen fertilization during the early stage and still maintained a high stomatal conductance and transpiration rate. As a result, the photosynthetic rate of V2 at the grain formation stage was much lower than that of V1, and the yield of V2 was significantly lower than that of V1, which is similar to the results of Liu et al. (2017). Therefore, appropriate water and nitrogen management (W_1_N_2_, W_2_N_2_) promotes leaf photosynthesis during the grain formation period and prolongs the duration for which leaves are green, ultimately achieving the goal of saving water and generating high yields for drip-irrigated rice.

### 4.3 Appropriate water and nitrogen management promotes nitrogen uptake and utilization and improves NUE

Previous research has indicated that water deficit reduces nitrogen uptake and assimilation by reducing the activity of key nitrogen metabolism enzymes (Ahanger et al., 2017). However, it has also been reported that the activity of key enzymes of nitrogen metabolism in wheat grains increases under high temperature, drought, and combined stress (Ru et al., 2022). In the present study, the GS content under W_1_N_2_ treatment was higher than that under W_2_N_2_ at the heading stage, but the NR content was lower than that under W_2_N_2_. GS is a key enzyme in the conversion of NH_4_^+^ into amides and amino acids. Under limited irrigation (W_1_), higher GS can fully assimilate NH_4_^+^ produced by NR catalysis and synthesize amino acids, which is conducive to the absorption of nitrogen at the heading stage (Zhang et al., 2022b). Additionally, low NR activity can rapidly promote the maintenance of osmotic pressure in photosynthetic cells by increasing the NO_3_^-^ level (Zhong et al., 2018); under deficit irrigation (W_2_), the increase in NR activity promotes the synthesis of NO, which in turn increases the sensitivity of stomata to ABA and is not conducive to the synthesis of photosynthetic products (Xing et al., 2021). High glutamine synthetase activity is a key reason for the improved NUE.

The uptake and assimilation of nitrogen by crops requires the participation of a series of nitrogen metabolic enzymes (Lam et al., 1996). These key enzymes of nitrogen assimilation and the final products of nitrogen metabolism are also the most important biochemical characteristics for improving NUE (Xu et al., 2012). Tian et al. (2018) showed that postponing topdressing and reducing the application amount of basal fertilizer can improve the efficiency of nitrogen assimilation and promote the uptake and accumulation of nitrogen, thereby achieving high yields. In this study, under the same water conditions, the postponement of nitrogen fertilization (N_2_) increased the NR and GDH contents of rice leaves from heading to 20 days after heading (**Figure 3**). These results indicate that under specific water conditions, the postponement of nitrogen fertilization can promote the uptake of nitrogen by rice leaves; the increase in NR and GDH activities also provides sufficient ammonium nitrogen for the nitrogen cycle; thus, the nitrogen demand can be met during the rice growth period, and the leaves can remain green for a longer time (Wu et al., 2018), which provides sufficient nitrogen for a high photosynthetic rate and physiological metabolism, thereby maintaining the yield and improving the NUE. It is worth noting that in this study, the postponement of nitrogen fertilization increased the GS content in V1 but reduced the GS content in V2 (**Figure 3**). This finding may be related to the different levels of drought resistance between cultivars. The reduction in the GS content may lead to the accumulation of NO_3_^-^ and NH_4_^+^, while NO_3_^-^ regulates the opening of stomata by affecting guard cells (Thomas and Hilker, 2000; Wilkinson et al., 2007). This approach may be a strategy for drought-sensitive rice (V2) to adapt to a water deficit, and the increase in the GS content is conducive to the rapid detoxification of NO_3_^-^ and NH_4_^+^ during the nitrogen metabolism of V1 (Thomas and Hilker, 2000). In addition, changes in enzyme activity can directly affect nitrogen uptake and NUE (Du et al., 2020). Therefore, in this study, the NUE of V1 was higher than that of V2.

**Figure 9:**
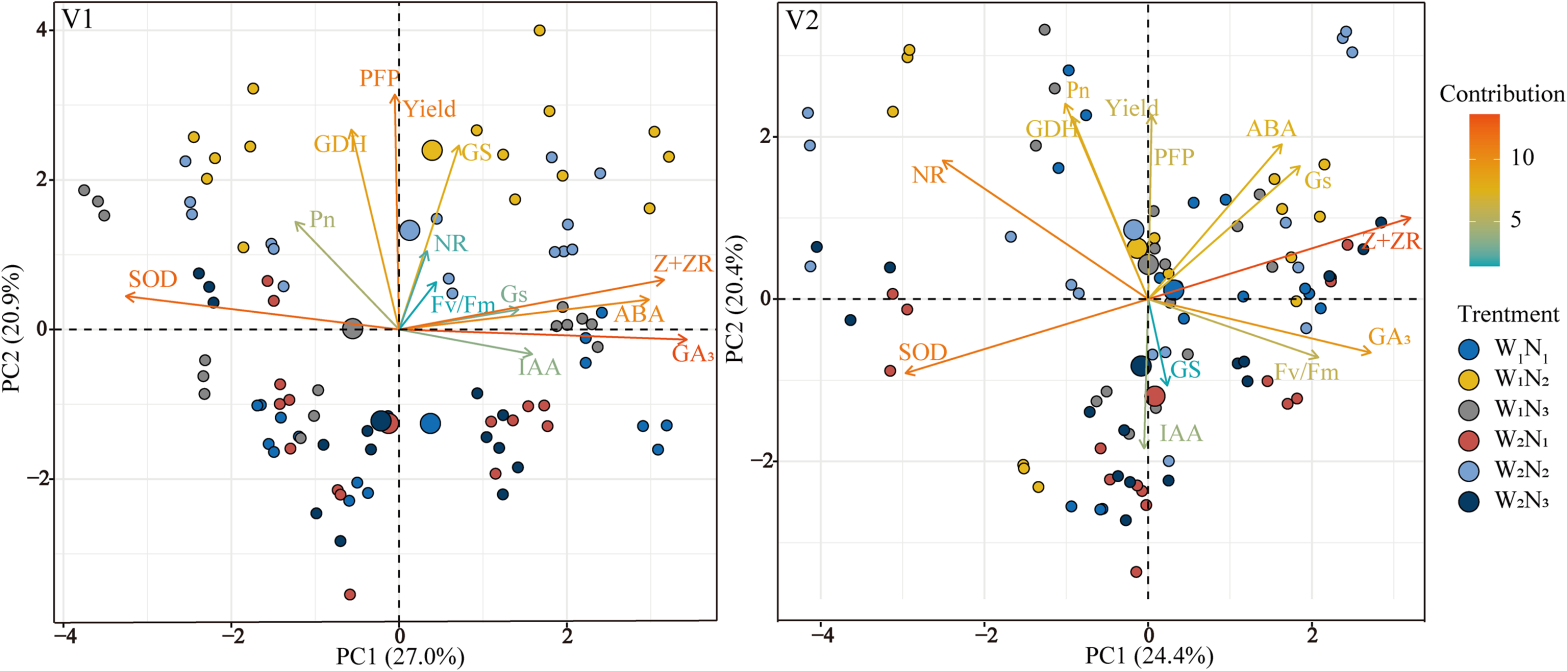
Principal component analysis of the main photosynthetic and physiological indices, yield, and NUE. *P*_n_, photosynthetic rate; *G*_s_, stomatal conductance; *F*_v_/*F*_m_, maximal photochemical efficiency of PSII; SOD, superoxide dismutase; NR, nitrate reductase; GS, glutamine synthetase; GDH, glutamate dehydrogenase, ABA, abscisic acid; IAA, indole acetic acid; GA_3_, gibberellic acid; Z+ZR, zeatin riboside. V1 and V2 represent T-43 and Liangxiang-3, respectively. PC, principal component.

The differences in the effects of water and nitrogen interactions on rice grain yield and NUE under drip irrigation can be explained by variations in gas exchange and nitrogen metabolism factors (**Figure 9**). Principal component analysis revealed that *P*_n_ and GDH were the most important physiological factors affecting rice yield performance, indicating that reasonable water and nitrogen management may promote optimal nitrogen transport capacity and photosynthetic efficiency, thereby increasing grain yields (Chen et al., 2018). Therefore, drip-irrigated rice maintained high activities of antioxidant enzymes and nitrogen metabolism enzymes at 20 days after heading to alleviate the growth stress caused by moisture reduction, thereby promoting the photosynthetic rate of the functional leaves during the grain formation stage and laying a foundation for full grain filling. This explanation may be the main reason for the high yield and NUE.

## 5 Conclusion

Under drip irrigation and plastic film mulching, the GDH and *P*_n_ under W_1_N_2_ were 153.4-930.3 and 19.2-49.7% higher, respectively, than those in the other treatments at 20 days after heading. W_1_N_2_ facilitates the uptake and utilization of N by functional rice leaves, delays leaf senescence, extends photosynthetic time, and promotes photosynthetic efficiency, thereby increasing yield and NUE. Compared with those of V2, the *P*_n_ and the contents of ABA, IAA, GA_3_, and ZR of V1 were higher at 20 days after heading. The yield was mainly closely related to *P*_n_ and GDH. In summary, a high yield and NUE can be achieved by cultivating the drought-resistant cultivar (T-43) under the appropriate water and nitrogen management mode (W_1_N_2_) for drip irrigation under plastic film mulching, which enhances the nitrogen metabolism activity of the functional leaves of drip-irrigated rice at 20 days after heading, promotes the uptake and utilization of nitrogen by leaves, improves the antioxidant enzyme activity, delays leaf senescence, increases the photosynthetic rate, and maintains the balance of endogenous hormones, realizing high production and efficient use of nitrogen fertilizer. This study contributes to our understanding of the biological water-saving potential of drip-irrigated rice in arid areas.

## Data availability statement

The original contributions presented in the study are included in the article/supplementary material. Further inquiries can be directed to the corresponding authors.

## Author contributions

YL and GW conceived and designed the experiment, and LZ performed the statistical analyses. LZ, QT, and ZS determined the physiological indicators and photosynthetic indicators. YL, GW, and YY critically reviewed the manuscript. All authors have read and approved the manuscript. All authors contributed to the article and approved the submitted version.
